# Generating Silicon Nanofiber Clusters from Grinding Sludge by Millisecond Pulsed Laser Irradiation

**DOI:** 10.3390/nano10040812

**Published:** 2020-04-23

**Authors:** Ko Momoki, Kunimitsu Takahashi, Kyosuke Kobinata, Yoshikazu Kobayashi, Akihito Kawai, Jiwang Yan

**Affiliations:** 1 School of Integrated Design Engineering, Graduate School of Science and Technology, Keio University, Hiyoshi 3-14-1, Kohoku-ku, Yokohama 223-8522, Japan; k_momoki_0821@z5.keio.jp; 2 DISCO CORPORATION, 13-11 Omori-Kita 2-chome, Ota-ku, Tokyo 143-8580, Japan; 3 Department of Mechanical Engineering, Faculty of Science and Technology, Keio University, Hiyoshi 3-14-1, Kohoku-ku, Yokohama 223-8522, Japan

**Keywords:** silicon nanofiber, laser processing, nanostructure, sludge waste, material reuse

## Abstract

Silicon nanofiber clusters were successfully generated by the irradiation of millisecond pulsed laser light on silicon sludge disposed from wafer back-grinding processes. It was found that the size, intensity, and growing speed of the laser-induced plume varied with the gas pressure, while the size and morphology of the nanofibers were dependent on the laser pulse duration. The generated nanofibers were mainly amorphous with crystalline nanoparticles on their tips. The crystallinity and oxidation degree of the nanofibers depended on the preheating conditions of the silicon sludge. This study demonstrated the possibility of changing silicon waste into functional nanomaterials, which are possibly useful for fabricating high-performance lithium-ion battery electrodes.

## 1. Introduction

Nowadays, a huge amount of silicon sludge is continuously produced during the slicing and grinding processes of single-/poly-crystal silicon wafers in the manufacturing of semiconductor devices and solar cells. Especially in the back-grinding of ultrathin silicon wafers for memory use, up to 90% of silicon material will be removed and disposed as sludge. However, it is extremely difficult to reuse the sludge for silicon ingot production as the sludge contains impurities such as diamond abrasive grains [1]. In recent years, attempts have been made to reuse the silicon sludge waste as a raw material for fabricating negative electrodes for lithium ion batteries (LiBs). In our previous work, for example, silicon sludge was mixed with carbon nanofibers and sintered as a network-structured thick film by nanosecond pulsed laser irradiation [2]. A mixture of silicon sludge with metal nanoparticles was sintered by a focused infrared light beam to generate porous and electrically conductive thick films on copper foils [3]. In addition, a nanosecond pulsed laser was used to process a mixture of silicon sludge and carbon black to generate silicon micro pillars on a copper foil, which showed ability for absorbing electrical charge-induced volume expansion of silicon and for performing as negative electrodes of LiBs [4].

However, the grain size of silicon sludge is quite big, averaging several microns. To further improve the performance of the silicon-based LiB electrodes, it is important to reduce the grain size of the silicon sludge, from micron level to 100 nm level or smaller [5,6]. There is an increasing requirement for silicon nanomaterials such as nanoparticles, nanofibers, and nanowires in the LiB industries [4,7,8,9]. These nanomaterials are currently produced from high-purity silicon powders/wafers by vapor–liquid–solid methods [10,11], or thermal/laser evaporation methods [12,13,14,15,16]. These methods require expensive raw materials and deposition systems for catalyst droplets, resulting in high production costs and additional impurities from the catalyst. Femtosecond pulsed laser ablation has been used for generating high-purity nanoparticles from pristine silicon wafers, but the production efficiency is low [17,18]. Reducing the grain size of silicon sludge by ball milling has also been attempted by some researchers, but it is extremely time-consuming and difficult to control the uniformity of particles on the nanometer scale [19,20].

Recent work by our research group demonstrated the possibility of generating uniform silicon nanoparticles from silicon sludge generated in multi-wire saw slicing [21] and wafer back-grinding [22] processes, by nanosecond laser irradiation. For both cases, the generated silicon nanoparticles were crystalline with an average size of ~10 nm [21,22]. Also, the impurity in silicon sludge, mainly diamond abrasives, could be vaporized and removed as carbon oxide during laser irradiation; thus, high-purity silicon nanoparticles were obtainable [21].

Compared with crystalline silicon nanoparticles, amorphous silicon nanoparticles might be more helpful for lithium ion transportation [23]. In addition, silicon oxide (SiO_x_) has been found to be useful in LiB electrodes because of its unique property whereby it plays the role of a buffer layer against large volumetric change during electrochemical cycling by forming reversible phases of LiO_2_ or Li_2_SiO_3_ [24,25]. Recently, silicon-catalyzed growth of amorphous SiO_x_ nanowires by continuous-wave laser ablation of SiO_x_ powders in high-pressure gas was achieved by Kokai et al. [26]. They found that in Ar or N_2_ gases, amorphous SiO_x_ nanowires with diameters up to 80 nm were grown, and the nanowires were attached with spherical crystalline Si nanoparticles at their tips and covered with thin amorphous SiO_x_ layers [26]. This type of nanomaterial may provide stable photoluminescence and excellent anode properties in LiB applications; thus, is receiving intensive attention from multidisciplinary research areas [27,28]. However, there is no available up to date literature on generating amorphous silicon nanoparticles or nanofibers with SiO_x_ composition from silicon sludge.

In this study, the possibility of generating amorphous silicon nanomaterials, namely nanofibers with SiO_x_ composition, from silicon grinding sludge by millisecond pulsed laser irradiation without the use of catalysts, was explored. The effect of laser pulse width, gas pressure, and sludge preheating on the size, morphology, and crystalline structure of the generated silicon nanomaterials were investigated, and the mechanism of laser-material interaction was clarified by high-speed camera observation of plume formation behavior. This study presents an environmentally friendly way to produce advanced silicon nanomaterials, which have extensive applications in energy storage devices and so on.

## 2. Materials and Methods

**Figure 1:**
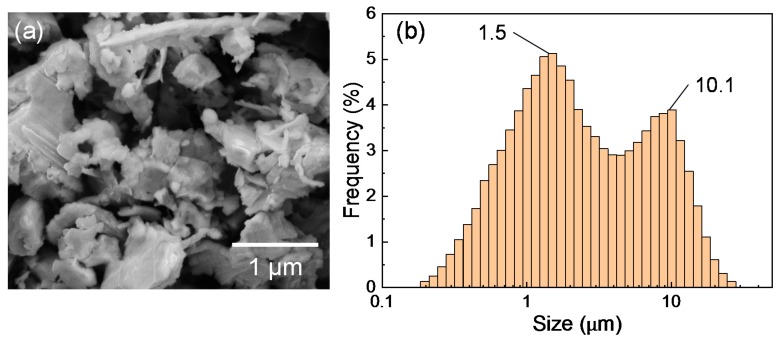
(**a**) SEM image and (**b**) size distribution of silicon sludge.

**Figure 2:**
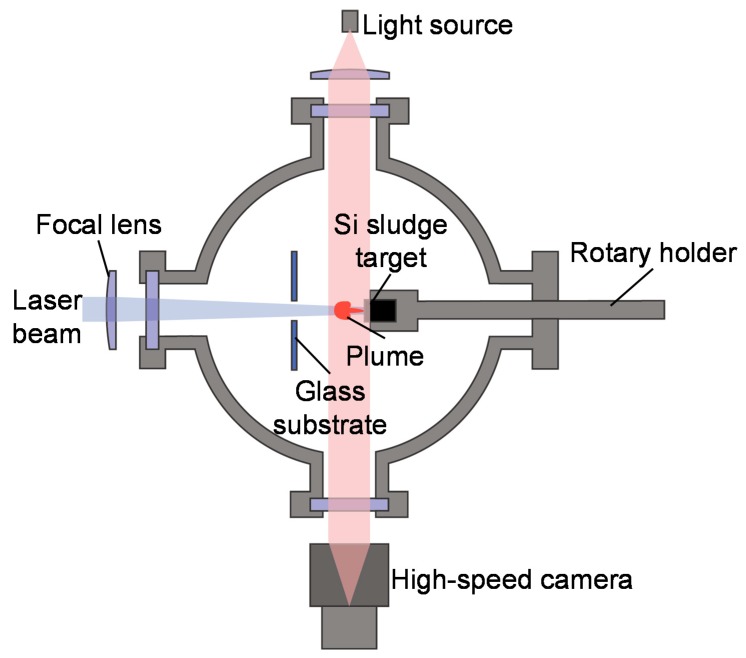
Schematic diagram of experimental setup.

The silicon sludge used in the experiments was produced from the back-grinding process of silicon wafers, which consists of two steps: rough grinding using resin-bond diamond wheels and fine grinding using vitrified-bond diamond wheels. Figure 1 shows the SEM photograph and size distribution of the sludge powder. There are two size peaks at 1.5 and 10.1 μm, respectively, corresponding to the two grinding steps; and the mean size is 3.9 μm. To prepare targets for laser irradiation, the silicon sludge was press molded into cylinders (diameter 10 mm, height 10 mm). A few targets were preheated at 400 and 900 °C in air for 1 h before laser irradiation to examine the possible effects of sludge oxidation. The targets were then placed in a chamber filled with Ar gas, as shown in Figure 2. The gas pressure was varied in the range of 1.3–101 kPa.

Laser irradiation was performed using a specially developed quasi-CW fiber laser with a wavelength of 1070 nm and a peak power of 500 W. The quasi-CW laser was pulsed by a pulse generator and the pulse width was varied from 1 to 10 ms. Laser irradiation was performed under a single-shot mode; thus, there was no pulse overlap. The laser beam had a diameter of 500 μm at the target surface and a Gaussian energy distribution with a power density of 178 kW/cm^2^. The generated silicon nanomaterials were backward transferred onto a glass substrate placed 50 mm away from the target. The nanomaterials were observed using two scanning electron microscopes (SEM), the Inspect F50 (FEI Company, Fremont, CA, USA) and the MERLIN Compact (Carl Zeiss AG, Oberkochen, Germany), for low and high magnifications, respectively, and characterized using a transmission electron microscope (TEM) Tecnai G2 (FEI Company, Fremont, CA, USA) with fast Fourier transform (FFT) analysis, and a laser micro Raman spectrometer NRS-3100 (JASCO Co., Tokyo, Japan). The laser-induced plume generation was observed by using a high-speed camera FASTCAM SA5 (Photron Ltd., Tokyo, Japan), and a shadowgraph image of the plume was obtained by using a light source placed behind the irradiated target. The plume generation was observed at a frame rate of 20,000 fps and a gate time (the time required to capture one frame of image) of 1/1,000,000 s, while the shadowgraph imaging was performed at 20,000 fps and 1/156,000 s, respectively.

## 3. Results and Discussion

### 3.1. Effect of Gas Pressure

**Figure 3:**
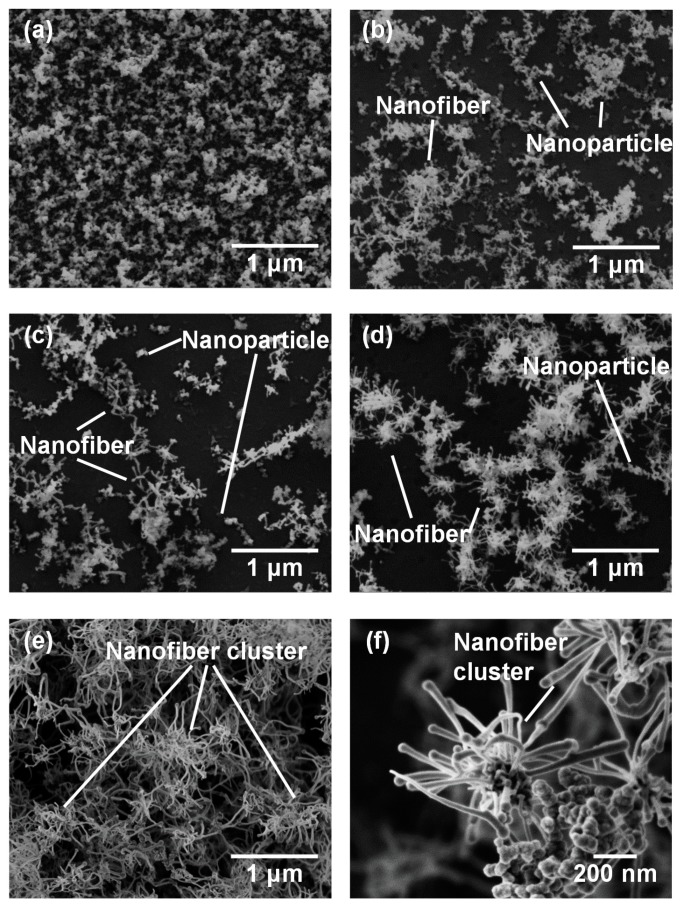
SEM photographs of nanomaterials generated at various gas pressures: (**a**) 1.3, (**b**) 13, (**c**) 40, (**d**) 67, and (**e**,**f**) 101 kPa.

**Figure 4:**
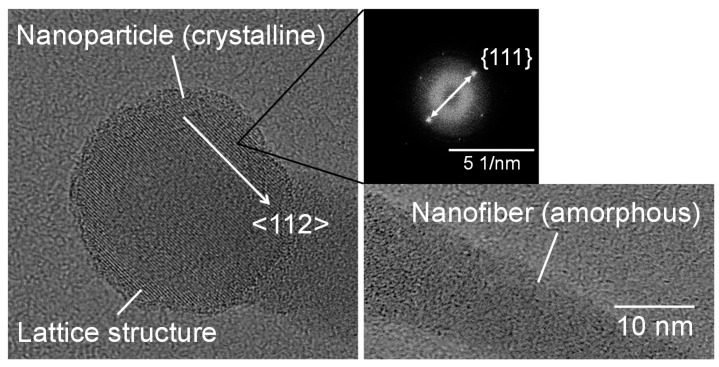
TEM images of a nanoparticle connected to a nanofiber generated at a gas pressure of 101 kPa.

Figure 3 shows SEM micrographs of the nanomaterials that were generated at different gas pressures at the laser pulse width of 10 ms. At 1.3 kPa, silicon nanoparticles were densely deposited on the glass substrate (Figure 3a). Between 13–67 kPa, silicon nanofibers were generated. The nanofiber to nanoparticle ratio increased with pressure (Figure 3b–d). At 101 kPa, nanofiber generation became dominant and the nanofibers were agglomerated into urchin-like clusters (Figure 3e) in which multiple nanofibers extended radially from the center of each “urchin”. Nanoparticles can be identified on the tips of the nanofibers (Figure 3f). Figure 4 is a TEM image of a nanoparticle with a nanofiber connected to it. The nanoparticle has a size of 23.7 nm and has a crystalline structure, whereas the nanofiber is 17.3 nm thick and has an amorphous structure. The FFT analysis shows the existence of {111} lattice planes with an interplanar distance of 3.14 Å in the nanoparticle, and shows the extension of the nanofiber along the <112> lattice direction.

**Figure 5:**
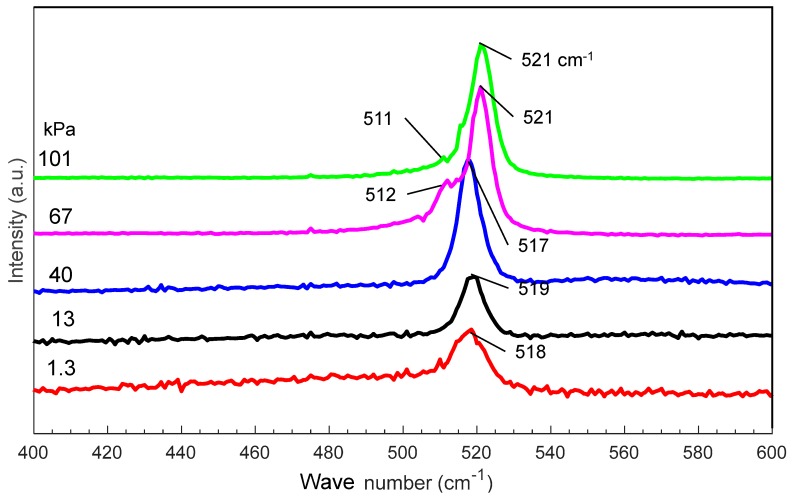
Raman spectra of silicon nanomaterials generated at different gas pressures.

Figure 5 shows the Raman spectra of nanomaterials generated at various gas pressures. As the pressure increases from 1.3 to 101 kPa, the main Raman peak shifts from 518 to 521 cm^−1^, indicating an increase in the average size of the nanomaterials. At 67 and 101 kPa, new peaks are observed at 511 and 512 cm^−1^, respectively, indicating the possible generation of nanocrystals in the amorphous nanofibers [29,30,31].

### 3.2. Effect of Laser Pulse Width

**Figure 6:**
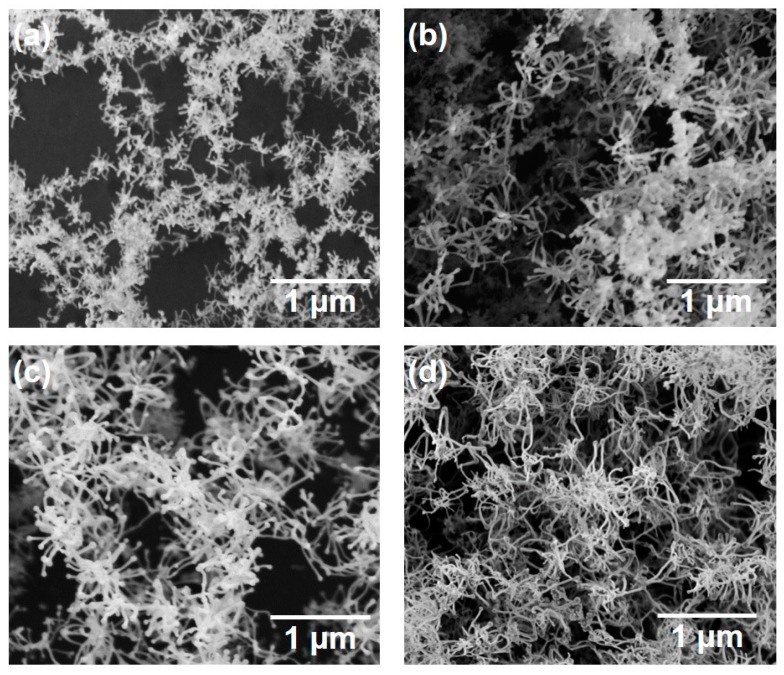
SEM photographs of silicon nanomaterials generated at various pulse widths: (**a**) 1, (**b**) 2, (**c**) 5, and (**d**) 10 ms.

**Figure 7:**
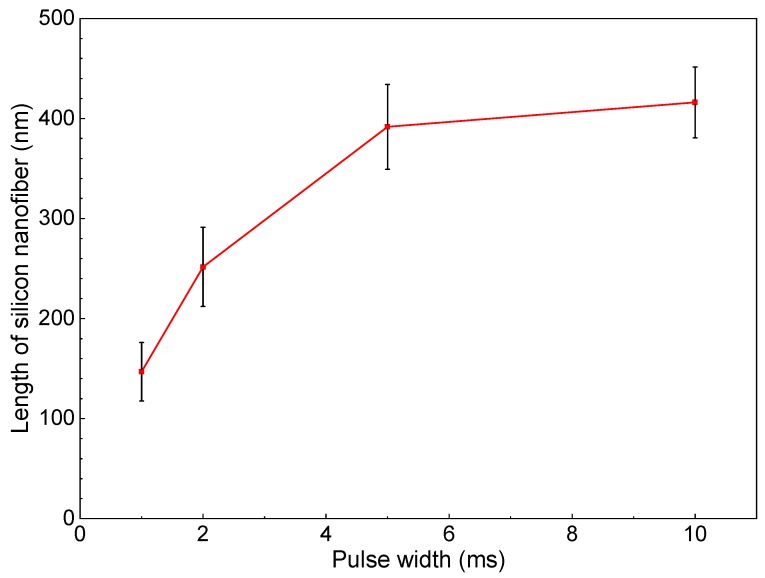
Change in length of silicon nanofibers with pulse width.

Figure 6 shows SEM photographs of nanomaterials generated at different pulse widths and the gas pressure of 101 kPa. The density of the deposited nanomaterials increased with the laser pulse width. Next, the average length of the nanofibers was extracted by an image processing program ImageJ and plotted in Figure 7. The average length of the nanofibers increased from 147 to 416 nm as the pulse width increased from 1 to 10 ms. It is postulated that the continuous supply of precursor material throughout the longer pulse contributed to the growth of longer nanofibers. Therefore, the nanofiber length and generation efficiency are controllable by the laser pulse width. As can be seen from the error bars in Figure 7, the dispersion of the fiber length variation is insignificant, indicating the stability of the results.

### 3.3. Effect of Sludge Oxidation Degree

**Figure 8:**
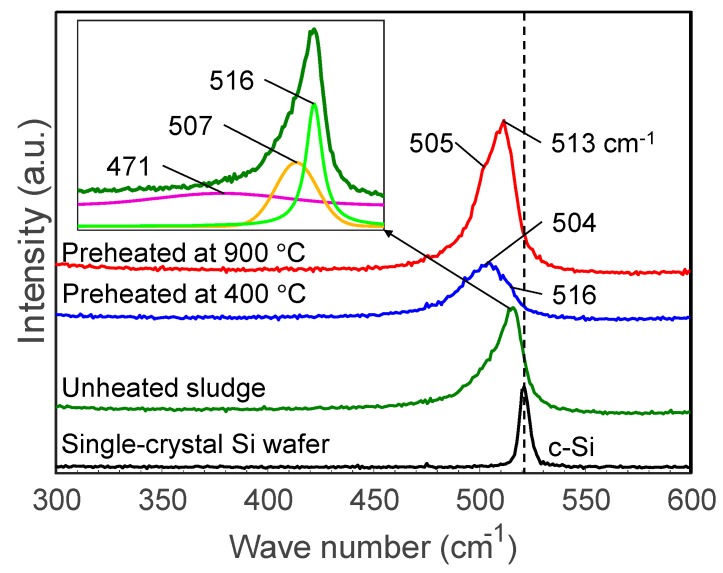
Raman spectra of sludge targets preheated at different temperatures. The insert shows an example of peak separation.

Figure 8 illustrates the Raman spectra of sludge targets preheated at different temperatures compared to the spectra of unheated sludge and a pristine silicon wafer. The unheated silicon sludge has an asymmetric broad peak which can be separated into peaks at 516, 471, and 507 cm^−1^, indicating that the grinding sludge is a mixture of nanocrystalline and amorphous silicon as well as SiO_x_. After heating, the peaks are broadened and the peaks around 504–505 cm^−1^ are enhanced, indicating that the heated sludge had been significantly oxidized.

**Figure 9:**
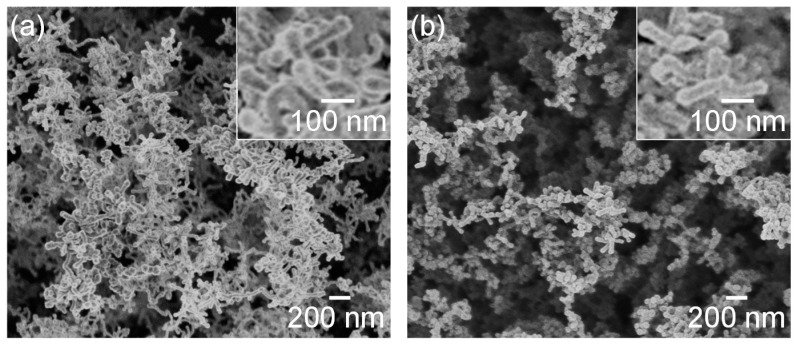
SEM photographs of nanomaterials generated from sludge preheated at (**a**) 400 and (**b**) 900 °C. The inserts are close-up views.

**Figure 10:**
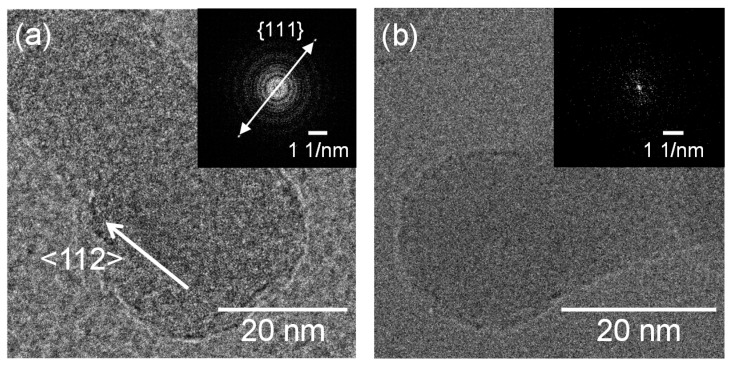
TEM images of silicon nanofibers generated from sludge preheated at (**a**) 400 and (**b**) 900 °C.

**Figure 11:**
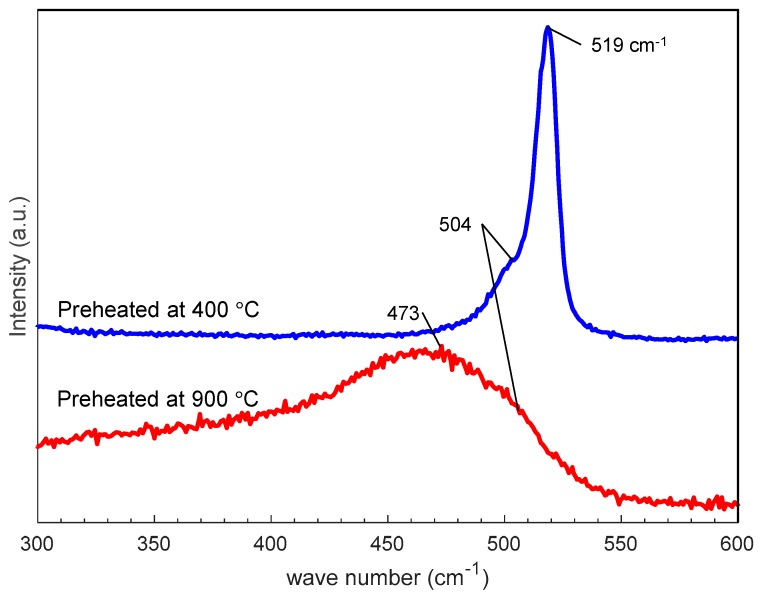
Raman spectra of nanofibers generated from silicon sludge preheated at different temperatures.

Figure 9 shows SEM photographs of nanomaterials generated from sludge preheated at 400 and 900 °C at the gas pressure of 101 kPa and pulse width of 10 ms. For both preheating temperatures, nanofibers were generated. The length of the nanofibers is shorter and the diameter thicker compared to the nanofibers generated from the unheated sludge (Figure 3e). This indicates that by preheating the target, the nanofiber growth was promoted along the radial direction and suppressed along the longitudinal direction. Figure 10 shows TEM images of nanomaterials generated from sludge preheated at different temperatures. In Figure 10a, the nanoparticle on the tip of the nanofiber has a crystalline core with {111} lattice planes, which have an interplanar distance of 3.03 Å, and the nanofiber runs along the <112> direction. This result is the same as that in Figure 4. However, in Figure 10b, a lattice structure is hardly observed while the FFT analysis shows only a halo ring, indicating that most parts of the nanomaterial are amorphous. Figure 11 shows the Raman spectra of the nanomaterials. By preheating the target at 900 °C, a broad peak was generated at approximately 470 cm^−1^, indicating that the generated nanomaterial has an amorphous structure. In contrast, crystalline silicon (519 cm^−1^) and SiO_x_ (504 cm^−1^) peaks are observed in the target preheated at 400 °C. This result indicates that the crystallinity of the nanomaterial can be controlled by the preheated temperature of the sludge, which can contribute to improve the capability of cathodes in lithium ion batteries, because amorphous silicon and SiO_x_ enhance their retention capacity [23,24,25].

### 3.4. Plume Generation Phenomenon

**Figure 12:**
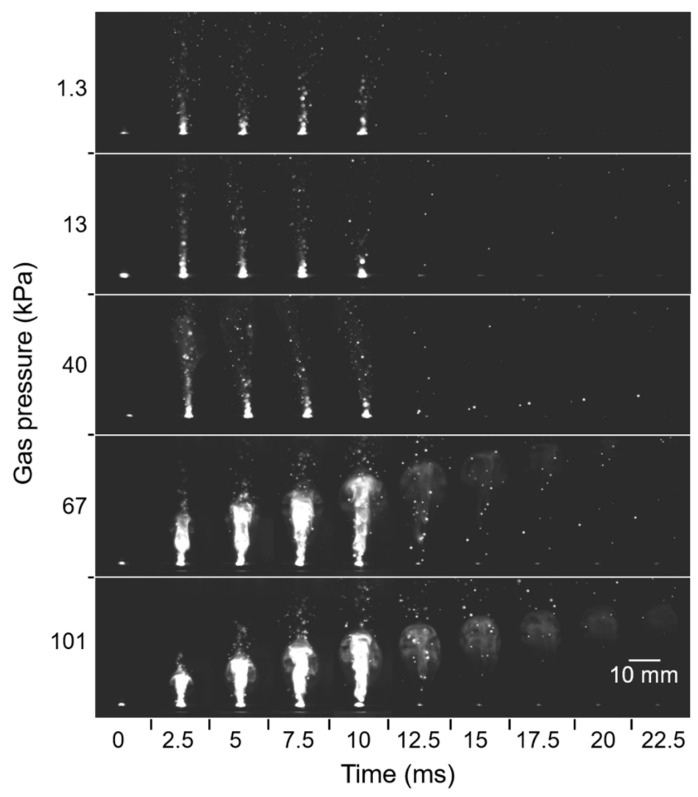
High-speed camera images of plume propagation at various gas pressures.

Plume generation and propagation during laser irradiation were observed, as presented in Figure 12, where the pulse width was 10 ms. As the Ar gas pressure increased, the size and intensity of the plume also increased, and the plume propagation lasted for a longer time. At gas pressures higher than 67 kPa, shining high-temperature columns of the plumes grew steadily with time and lasted even after the duration of the laser pulse. At pressures lower than 40 kPa, however, the high-temperature columns of plumes did not grow, and they disappeared right after the duration of the laser pulse. Instead, extremely small droplets were scattered toward the glass substrate. These differences were caused by the effect of pressure on plume expansion [32]. At low pressures, the propagating plume had a long mean free path, resulting in rapid diffusion of the plume and subsequent reduction of plume density. In contrast, at higher pressures, the smaller mean free path restricted plume diffusion. The spatial confinement led to stable growth of the high-temperature plume over a long duration without a sudden reduction of plume intensity. This scenario enabled continuous nanofiber growth from the nanoparticle cores.

**Figure 13:**
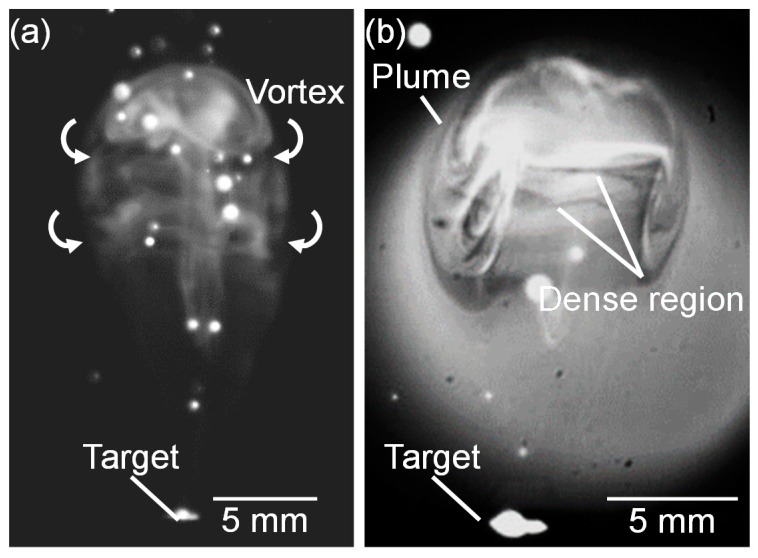
(**a**) Magnified high-speed camera images of plume propagation captured at 13.4 ms and 101 kPa, and (**b**) corresponding shadowgraph.

Figure 13 shows a high-speed camera image of a propagating plume at 13.4 ms after laser pulse irradiation at the gas pressure of 101 kPa, and a corresponding shadowgraph. In the plume, dense vortexes were formed in the vertical direction to the plume propagation (Figure 13a). It is presumed that the silicon atoms in the propagating plume collide with ambient gas molecules. The collided layer stays at the outer side of the plume forming vortexes in which a high-temperature and a high-density atom distribution is generated, as shown in Figure 13b.

### 3.5. Mechanism of Nanofiber Cluster Formation

**Figure 14:**
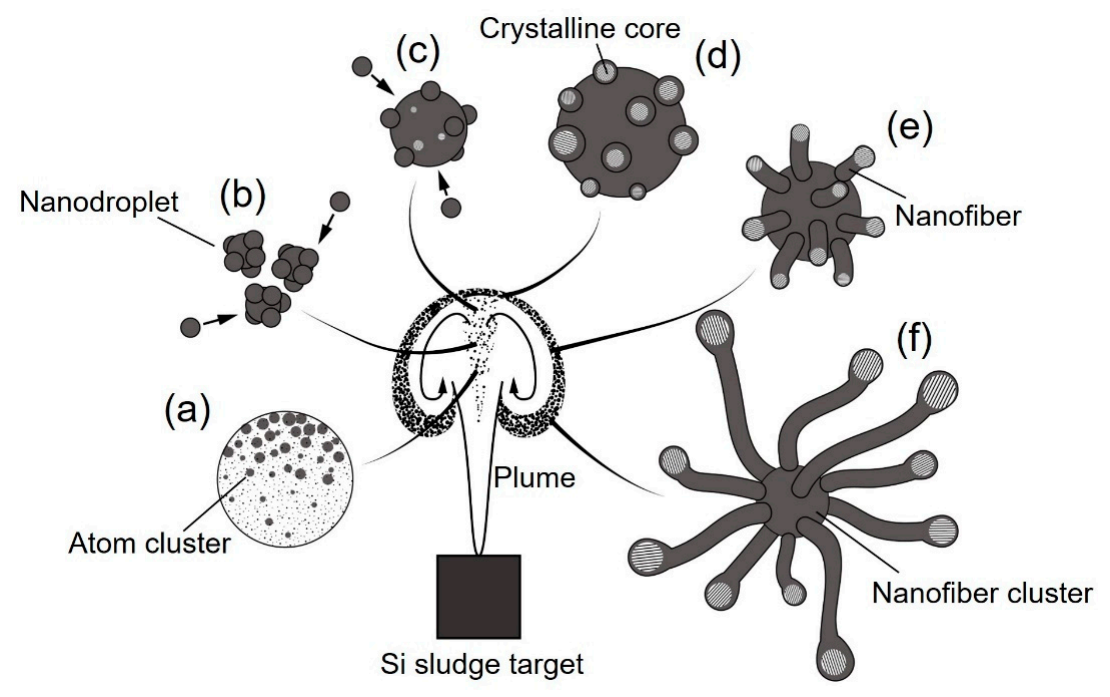
Schematic diagram of the mechanism of urchin-like silicon nanofiber cluster formation in a dense vortex: (**a**) atom clustering, (**b**) nanodroplet generation, (**c**) nanodroplet growth, (**d**) crystalline core nucleation, (**e**) nanofiber growth, and (**f**) nanofiber cluster formation.

Urchin-like nanofiber clusters are a potential nanomaterial for super high-performance lithium ion battery electrodes, because they act as a buffer layer due to the excellent resiliency of 1D nanostructured materials [23]. Figure 14 is a schematic diagram of the urchin-like silicon nanofiber cluster formation mechanism. The formation of a dense vortex during plume propagation is essential for nanofiber growth. Inside the plume vortex, crystalline cores are generated from atom clusters, nanodroplets, and their further aggregation (Figure 14a–c). The crystalline cores grow by absorbing amorphous silicon and oxygen, which come from the oxide layers of the sludge powders, and reduce the dangling bonds to lower the surface energy (Figure 14d). The amorphous layers further grow into nanofibers along the <112> lattice direction (Figure 14e). Among the various lattice surfaces, the {111} surface has the lowest surface energy. Crystalline cores grow easily along the <112> direction because of the reduction of the total surface energy [33]. Through the self-catalyst material growth, urchin-like nanofiber clusters are generated during cooling and deposited on the glass substrate (Figure 14f). To facilitate the nanofiber cluster generation, a high gas pressure is necessary to enable the propagating plume to collide with the gas molecules and form high-temperature dense vortexes. In addition, a long pulse width is essential for providing a sufficiently long time span for nanofiber cluster growth.

## 4. Conclusions

Millisecond pulsed laser irradiation was performed on silicon grinding sludge. At low gas pressures, silicon nanoparticles were generated. Urchin-like silicon nanofiber clusters were generated at high gas pressures. Silicon nanofibers grew along the <112> lattice direction from crystalline phase nanoparticle cores. A longer pulse width resulted in longer nanofibers. Moreover, preheating the sludge at a high temperature promoted amorphous phase formation and silicon oxidation in the nanofibers. High-speed camera observations confirmed the generation of dense vortexes in the plumes, which accommodated and provided spatial and temporal conditions for self-catalyst silicon nanofiber growth and urchin-like nanofiber cluster formation. This study demonstrated the possibility of a cost-effective method for generating silicon nanomaterials from wafer back-grinding sludge, which contributes to material resource reuse, sustainable manufacturing of LiBs, and future industrial symbiosis.
